# DNA methylation in Hepatoblastoma-a literature review

**DOI:** 10.1186/s13052-020-00877-6

**Published:** 2020-08-05

**Authors:** Gang Shen, Hongyu Shen, Jing Zhang, Qingtao Yan, Huixian Liu

**Affiliations:** 1 Pediatric Surgery Department, Weifang Peoples’ Hospital, Weifang, China; 2 Ultrasound Department, Weifang Haifushan Hospital, Weifang, China; 3 Dermatology Department, Weifang Peoples’ Hospital, No. 151, Guangwen Street, Kuiwen District, Weifang, 261041 China

**Keywords:** Methylation, Hepatoblastoma, DNMTs, TETs, UHRF

## Abstract

Hepatoblastoma (HB) is the most common malignant liver tumor in children. Abnormal activation of the Wnt/β-catenin signaling pathway plays an important role in the formation and development of HB. Genes in HB show a global hypomethylation change, accompanied by hypermethylation of specific tumor suppressor genes (TSGs). This article reviews the hypermethylation changes in several TSGs, such as RASSF1A, SOCS1, APC, HHIP, and P16, and analyzes the pathways and mechanisms of TSGs regulating gene expression. The role of the methylation-regulating enzymes DNA methyltransferases (DNMTs) and ten-eleven translocation (TET) family members enzymes in the methylation changes of HB was analyzed, and it was speculated that the occurrence of HB is partly due to the obstruction of liver differentiation in the early stage of differentiation. The origin cells may be incompletely differentiated hepatocytes remaining in the liver of children after birth. Therefore, further studying the role of methylation regulating enzymes in methylation changes in HB is a promising future research direction.

## Background

Hepatoblastoma (HB) is the most common malignant liver tumor in children, accounting for more than 80% of all pediatric malignant liver tumors and 1% of all pediatric tumors. The incidence rate is 1.5 ppm, and the annual growth rate is 2.7% [1, 2]. The histological types of HB are diverse but mainly include epithelial cell types: pure fetal type, embryonic type, mixed type, and small-cell undifferentiated type. The varieties of different histological types suggest that the tumor cells are in different stages of differentiation and that the clinical characteristics are different. HB may originate from primary hepatoblasts or from less differentiated liver stem cells or human fetal liver multipotent progenitor cells (hFLMPCs) [3]. A meta-analysis revealed similarities between HB, early mouse embryonic liver, and hepatocyte-derived stem/progenitor cells, suggesting that HB may originate from hepatic precursor cells [4]. HB cells can express CD44, CD90, CD133, and other cancer stem cell markers, suggesting that cancer stem cells also exist in HB [5].

The specific mechanism for the transformation of normal hepatocytes into HB is unknown. Cells may begin to transform in an early stage of liver differentiation, or the transformation may be caused by gene mutation after liver development completed. The Wnt/β-catenin signaling pathway plays a key role in the process of tumorigenesis. The catenin beta 1 gene (CTNNB1) encodes the β-catenin protein, and mutations in CTNNB1 occur widely in HB. Mutations in CTNNB1 are closely related to HB and are most likely the original aberrations leading to tumorigenesis. In normal hepatocytes, the β-catenin N-terminal serine and threonine residues encoded by CTNNB1 exon 3 are phosphorylated by the APC regulator of WNT signaling pathway/Axin/ glycogen synthase kinase 3 beta (APC/Axin/GSK3β) complex and then recognized by the ubiquitin-conjugating complex and ubiquitinated. The protein is subsequently degraded by the proteasome, which reduces the β-catenin protein concentration in the cytoplasm and inactivates the Wnt/β-catenin signaling pathway [6]. Mutation of the CTNNB1 gene causes the β-catenin protein to be unable to be degraded, abnormally aggregate in the cytoplasm, and ectopically transferred to the nucleus [7]. In the nucleus, β-catenin associates with the T cytokine/lymphocyte enhancement factor binding factor (TCF/LEF) family. Transcription factors bind and recruit coactivators (BCL9, CBP/300, Pygo, etc.) to activate the transcription of downstream target genes [8]. Approximately 60–80% of HBs have CTNNB1 mutations, including point mutations and deletions in exon 3 [9]. Other genes with mutations in HBs include APC (20.51%), AXIN1 (1.67%), AXIN2 (3.75%), and leucine-rich repeat-containing G protein-coupled receptor 6 (LGR6) (12.5%) [10].

In the 1980s, Feinberg and Vogelstein first linked the epigenetic inheritance of genes to cancer [11]. Epigenetics alters the spatial structure of genes by modifying genes involved in processes such as DNA methylation, histone deacetylation, and RNA methylation, regulating gene transcription and translation and affecting gene expression. The expression of proteins is different between different organs, and the protein expression within the same organ is different at different developmental stages. Epigenetics plays an important role in this process. In the past few decades, an increasing number of studies have shown that epigenetic changes are widely involved in the occurrence, invasion, and metastasis of cancer [12] and promote tumorigenesis and tumor development by inactivating tumor suppressor genes (TSGs) and activating oncogenes. HB gene mutations are simple, and whole-exon sequencing shows that there are only 2.9 mutations per HB tumor on average [13], which is much lower than 35–66 mutations per tumor in liver cancer [14–16], indicating that HBs are not only caused by the accumulation of gene mutations; in addition, most HBs occur in children, suggesting that epigenetic mechanisms that control development play an important role in the occurrence and development of HB. This article reviews the role of DNA methylation in HB occurrence, diagnosis, and treatment.

## Changes in DNA methylation in HB

HB is a rare tumor, and there is sparse literature on DNA methylation. In the PubMed database, only 97 articles were returned from a search using the keywords “hepatoblastoma” and “methylation”, which is far lower than the 2513 articles returned using the keywords “hepatocellular carcinoma” and “methylation”. This shows that methylation has been far less studied in HB than in other tumors. There are a large number of differentially methylated regions (DMRs) and differentially methylated sites (DMSs) in HB and normal liver cells. Using the HM450K methylation chip to analyze HB tissue and adjacent normal liver tissue, 1359 DMSs were detected, involving 765 genes. Of these DMSs, 58% were hypomethylated, and most of the hypermethylated sites were located in the CpG islands in the promoter region of the genes. These 1359 CpG sites overlapped highly with CpG sites that were altered during liver development, suggesting that some of the HBs were caused by methylation changes in the early stages of liver development [17]. According to GO and KEGG analyses of the different genes, the three most affected physiological processes were cell adhesion, blood coagulation, and nervous system development, and the three most affected cellular functions were protein binding, nucleotide binding and ATP binding [18]. The lower overall methylation levels in HB cells than in normal hepatocytes have also been confirmed by other experiments [18]. These data show that methylation in HB is characterized by low levels of global DNA methylation and high levels of methylation of specific genes, which is consistent with the methylation characteristics of other tumors [19].

DNA methylation changes, such as hypomethylation of oncogenes and hypermethylation of TSGs, regulate gene expression, acting as an inactivator of TSGs and an activator of oncogenes [20]. The regulation of imprinted genes involved in methylation also plays a role in tumorigenesis in HB [21]. DNA methylation regulates gene expression and affects the expression of downstream genes through various pathways, playing a role in inhibiting cancer cell apoptosis, promoting cancer cell proliferation and cell cycle dysfunction, and promoting tumor metastasis (Table 1).

**Table 1:** Changes in DNA methylation and the effects on HB

Gene	Methylation status of promoter	Possible mechanisms	Effects on HB
RASSF1A	hypermethylation	Hippo pathway	metastasis; poor responsiveness to chemotherapy; With poor prognosis;
SOCS1 [22–24]	hypermethylation	JAK/STAT pathway	Tumor formation and growth; Liver regeneration
APC [25]	hypermethylation	Wnt/β-Catenin pathway	Tumorigenesis
HHIP [26, 27]	hypermethylation	Hh pathway	Tumor growth; Angiogenesis;
P16	hypermethylation	Cell cycle	Tumorigenesis or no effect
MT1G	hypermethylation	PI3K/Akt pathway	With poor prognosis
NDRG2 [28]	hypermethylation	ERK1/2 pathway;	With poor prognosis; Metastasis
GPR180	hypermethylation	Unknown	Metastasis
MST1R	hypermethylation	Unknown	Metastasis
OCIAD2 [29]	hypermethylation	AKT pathway	metastasis; Tumor growth and invasion
PARP6	hypermethylation	Unknown	metastasis
AFP	hypomethylation	Hippo pathway	Diagnostic biomarker; Prognostic biomarker; Follow-up indicator; Chemotherapy responsiveness indicator
KLK4	hypomethylation	Unknown	Tumorigenesis
MYCN	hypomethylation	Unknown	Tumor growth
PLAG1	Loss of imprinting	IGF2 pathway	With poor prognosis; Tumorigenesis
H19/IGF2	Loss of imprinting	IGF2 pathway	Tumorigenesis
IGFBP3	hypermethylation	IGF2 pathway	Tumorigenesis

### DNA methylation as an inactivator of TSGs

At present, DNA methylation in HB has been found mainly to regulate the expression of TSGs, and many TSGs have been studied.

**Fig. 1:**
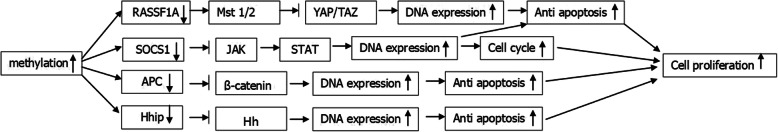
Increased DNA methylation results in decreased expression of multiple genes, increased cell proliferation through different pathways, and caused tumor formation or metastasis

Ras association domain family member 1A (RASSF1A) is a widely studied TSG that is inhibited and inactivated in lung, breast, kidney, bladder and ovarian cancers, and DNA methylation plays an important role in gene inactivation [30]. In HB, the methylation rate of the RASSF1A gene is 33.8 to 44.3%, and RASSF1A gene methylation is directly related to distant metastasis and poor prognosis [31, 32]. The incidence of RASSF1A gene methylation is high, and the overall survival rate and event-free survival rate associated with high RASSF1A gene methylation are lower than those associated with low RASSF1A gene methylation [33]. Compared with patients with low methylation levels of the RASSF1A gene, patients with high methylation levels of the RASSF1A gene have a high incidence of distant metastasis and a low overall survival rate and event-free survival rate [31–33]. RASSF1A promoter methylation also predicts low responsiveness to chemotherapy [33]. Methylation of the promoter region of the RASSF1A gene silences the gene and inhibits the macrophage stimulating 1/2 (Mst1/2) protein, thereby activating the core protein Yes-associated protein/Tafazzin (YAP/TAZ) of the Hippo pathway; this inhibits the apoptosis of cancer cells and promotes the proliferation of cancer cells [34, 35], and the Hippo pathway plays an important role in the formation of HB [36] (Fig. 1).

The suppressor of cytokine signaling 1 (SOCS1) gene has a dual opposite, it can function as either a tumor suppressor or a tumor promoter. SOCS1 gene was overexpressed in human breast cancer, epidermis and neuronal tumors and showed the characteristics of an oncogene [37–39]. Studies have shown that in HB, hypermethylation in the CpG region of the SOCS1 gene promoter leads to decreased SOCS1 gene expression in nearly half of the cases and decreased levels of the suppressor of cytokine signaling 1/ Jun activating binding protein 1/ STAT-induced STAT inhibitor-1 (SOCS1/JAB1/SSI-1) protein complex, activating the the Janus kinase-signal transducers and activators of transcription (JAK/STAT) signaling pathway and inhibiting apoptosis of cancer cells [40]. Other results also showed that the methylation level in the promoter region of the SOCS1 gene in HB increased [33, 41], indicating that abnormal DNA methylation increases SOCS1 silencing and plays an important role in the development of HB (Fig. 1).

In the canonical Wnt/β-catenin pathway, the degradation of β-catenin protein depends on the function of APC, Axin1/2, casein kinase 1 (CK1), and GSK3 protein complexes [42]. A decrease in APC promotes HB development by reducing the degradation of β-catenin through two mechanisms. One mechanism is mutation or deletion [43], as when the APC gene is mutated, APC cannot play its functional role, and the degradation of β-catenin protein is reduced. The second mechanism involves DNA hypermethylation, which inhibits the expression of APC and causes the APC gene to lose its function [41]. Familial adenomatous polyposis (FAP) is mainly caused by mutations in the APC gene, and the incidence of HB in patients with FAP is greatly increased [44] (Fig. 1).

The Hedgehog (Hh) pathway plays an important role in regulating the development of endoderm-derived tissues during embryogenesis. When embryonic development is complete, Hedgehog-interacting protein (HHIP) inhibits the Hh pathway, leading to the disappearance of or a reduction in Hh signaling pathway activity [45]. The hypermethylation of the HHIP gene promoter region results in nonexpression of HHIP protein; as such, the inhibition of the Hh pathway disappears, and the abnormal activation of the Hh pathway leads to the occurrence of various tumors [46, 47]. In HB, the HHIP gene promoter region is hypermethylated in 26% of patients [48]. In in vitro experiments, treatment of HB cell lines with the DNA demethylating drug 5-azacytidine restored HHIP protein expression, increased tumor cell apoptosis, and reduced tumor activity, indicating that abnormal activation of the Hh pathway caused by hypermethylation of the HHIP gene plays an important role in the occurrence and development of HB [48] (Fig. 1).

The P16 gene (CDKN2/MTS-1/INK4A) is a TSG that regulates the cell cycle, and its transcription product, P16 protein, is an inhibitor of cyclin-dependent kinases 4 (CDK4) and 6 (CDK6) [49]. It has been proven that in many tumors, methylation of the promoter region of the P16 gene leads to nonexpression of the P16 gene, which is involved in tumor formation [50]. Whether the P16 gene methylation is involved in HB formation is still controversial. Studies have reported abnormal methylation in the P16 gene promoter region in 12 of 24 HB patients, with significantly reduced P16 protein expression, suggesting that the P16 protein is involved in the tumorigenesis of HB [51]. Some scholars also performed methylation-specific PCR (MSP) and found that there was no hypermethylation of the P16 gene promoter in 27 HB tissues, and changes in the CDKN2A gene family were not related to the occurrence of HB [52, 53]. The role of the P16 gene in HB needs further experimental confirmation.

There are other TSGs involved in HB formation due to DNA methylation. Methylation levels in the metallothionein-1G (MT1G) gene promoter region were specifically increased, and hypermethylation in the MT1G promoter region was directly associated with poor prognosis, suggesting that MT1G could be used as a diagnostic marker and a potential drug therapeutic target [41]. The N-Myc downstream regulated gene 2 (NDRG2) gene plays the role of the TSG in many tumors [54], and its promoter region in HB presents hypermethylation, which is directly related to the prognosis of tumors [41]. Hypermethylation of the genes G protein-coupled receptor 180 (GPR180), macrophage stimulating 1 receptor (MST1R), OCIA domain containing 2 (OCIAD2), and poly (ADP-ribose) polymerase family member 6 (PARP6) was also involved in the tumorigenesis of HB, and methylation status was significantly correlated with metastasis [55].

### DNA methylation is an activator of oncogenes

DNA methylation in HB is characterized by decreased global methylation levels, weakened inhibition of oncogenes, increased expression of oncogenes, and promoted tumor proliferation. However, there are few studies of oncogene methylation in HB. There are many types of proteins with increased expression in HB, such as glypican 3 (GPC3) [56], transforming growth factor-β2 (TGF-β2) [57], CBP/P-300 interacting transactivator 1 (CITED1) [58], nuclear receptor subfamily 5 group A member 2 (LRH-1, NR5A2) [59], PCNA clamp associated factor (NS5ATP9, PCLAF) [60], and serpin family B member 3 (SERPINB3) [61], but most of the literature has focused on the effect of increased protein expression on tumors. The reason for the increase in protein expression is less studied. Nevertheless, it is certain that the increase in some proteins is related to the reduced methylation of related genes (Table 1).

Alpha-fetoprotein (AFP) is the most commonly used marker for the diagnosis and prognosis of liver cancer and HB. Its level is directly related to clinical prognosis and is an important basis for clinical staging. AFP also plays an important role in liver cell growth, differentiation, angiogenesis, apoptosis, and immune regulation [62]. Some experimental results have pointed out that the methylation level of the AFP gene promoter region in HB cells is significantly lower than that in normal liver cells, and the mRNA expression is increased [18], which indicates that gene methylation plays an important role in the upregulation of AFP expression (Table 1).

The kallikrein-related peptidase 4 (KLK4) gene is considered an oncogene and plays an important role in the formation of many tumors, such as gastric cancer and prostate cancer [63]. Studies have shown that in HB, the methylation level of the gene promoter region is reduced, and the expression of KLK4 mRNA and protein is increased [64]. Gene methylation plays an active role in oncogene expression. The MYCN gene is also an important oncogene and plays an important role in the formation of pediatric neuroblastoma and medulloblastoma [65]. In pediatric HB, MYCN is overexpressed, and its promoter region is hypomethylated. The use of the MYCN inhibitors alisertib (MLN8237) and JQ1 can inhibit tumor growth, indicating that the MYCN gene is also involved in the tumorigenic process in HB [66] (Table 1).

### Regulation of DNA methylation of imprinted genes

Imprinted genes play an important role in tissue development and differentiation. DNA methylation is an important way to maintain gene imprinting. Loss of imprinting (LOI) caused by changes in methylation is involved in the formation of various tumors.

The human chromosomes include more than 30 imprinted regions. In HB, one study used 12 cases of HB and adjacent normal liver tissues and found that all tumors were abnormally methylated in at least one DMR [21]. Of the 33 DMRs studied, 18 showed abnormal methylation, while 15 did not show abnormal methylation. There is hypomethylation in liver tissues adjacent to the tumor; both hypermethylation and hypomethylation are present in the tumor tissue, and hypermethylation is more frequent. It is speculated that prior to tumorigenesis, some DMRs in adjacent normal liver tissues are hypomethylated. This hypomethylation may be a common phenomenon in embryonic tumors. Hypermethylation only occurs in tumor tissues and may be obtained during tumorigenesis [21]. The most common sites for gene and epigenetic changes are 11p15.5 and 20q13.3. H19 and IGF2, which are closely related to tumors, are located in the 11p15.5 region [21, 67].

Pleomorphic adenoma gene 1 (PLAG1) is a gene located on 8q12.1, and its expression product is a transcription enhancer of the P3 enhancer of the insulin-like growth factor 2 (IGF2) gene. Studies showed that the PLAG1 gene was highly expressed in 19/20 cases of HB, which is 3–12 times higher than the normal liver expression level of children of the same age and similar to that in the embryonic stage, and his high expression is directly related to the poor prognosis of HB [68]. This result shows that the PLAG1 protein promotes tumorigenesis by enhancing the transcription of the H19/IGF2 genes.

H19/IGF2 are currently the most widely studied imprinted genes and play a key role in the development of many tumors. In normal cells, the H19 gene is maternally expressed and paternally imprinted, and the IGF2 gene is paternally expressed and maternally imprinted. The expression of H19 and IGF2 genes is affected by the differentially methylated region (DMR) upstream of H19 and the downstream enhancer of H19 [69]. If the methylation of the DMR region is abnormal, then H19 or IGF2 suffers the LOI, which will lead to the occurrence of many tumors, including HB [70]. The methylation status of the H19 differentially methylated region (DMR), the loss of heterozygosity (LOH), and the expression of the IGF2 allele in 54 HB tumors was analyzed, and the results showed that 12 cases (22%) had LOH, 9 cases (17%) had loss of imprinting (LOI) [71], and IGF2 was overexpressed in HB. These findings indicate that disruption of the H19/IGF2 gene imprinting plays an important role in the formation of some HBs.

Insulin-like growth factor-binding protein 3 (IGFBP3) is a multifunctional protein synthesized by the liver. IGFBP3 functions through the H19/IGF axis. It mainly mediates growth inhibition and induces apoptosis by binding to IGF [72]. Studies have shown that the IGFBP3 gene is highly expressed in normal liver tissue and is low in HB, mainly due to hypermethylation of the promoter region [73]. Treatment of HB cell lines with 5-aza-2-deoxycytidine demethylated the promoter region, resulting in reduced cell colony formation and a reduced ability to invade and metastasize. It was also found that hypermethylation of the IGFBP3 promoter region was related to macrovascular invasion and distant metastasis [73].

### Abnormalities of DNA methylation regulatory enzymes in HB

DNA methylation regulatory enzymes mainly include DNA methyltransferases (DNMT1, DNMT2, DNMT3A, DNMT3B, and DNMT3L) and demethylation enzymes (TET1, TET2, and TET3). DNA methyltransferases (DNMTs) increase DNA methylation and ten-eleven translocation (TET) family members enzymes play a demethylating role, reducing DNA methylation levels.

In HB, studies have found that DNMT1, DNMT3A, UHRF1, TET1, and TET2 are all upregulated and 5hmC is increased [74]. The DNMT1/UHRF1 complex is key for methylation maintenance. Increased expression of DNMT1, DNMT3A and UHRF1 proteins can cause hypermethylation of the gene promoter region, which may play a key role in the hypermethylation of the promoter region of HB TSGs [74]. Other experiments have also confirmed that UHRF1 expression in HB is increased [75], TSG promoter region methylation is increased, and gene expression is decreased. After knockout of UHRF1, TSG was re-expressed, indicating that UHRF1 is a key protein promoting hypermethylation and inhibiting the expression of TSGs, which can lead to hypermethylation of TSGs and inhibit expression. There are also data showing that the overexpression of UHRF1 damages the correlation between UHRF1 and DNMT1 [76], making DNMT1 unstable and ectopic [77], and inhibiting the activity of DNMT3A [78], which may also be an important reason for the global hypomethylation of tumors.

**Fig. 2:**
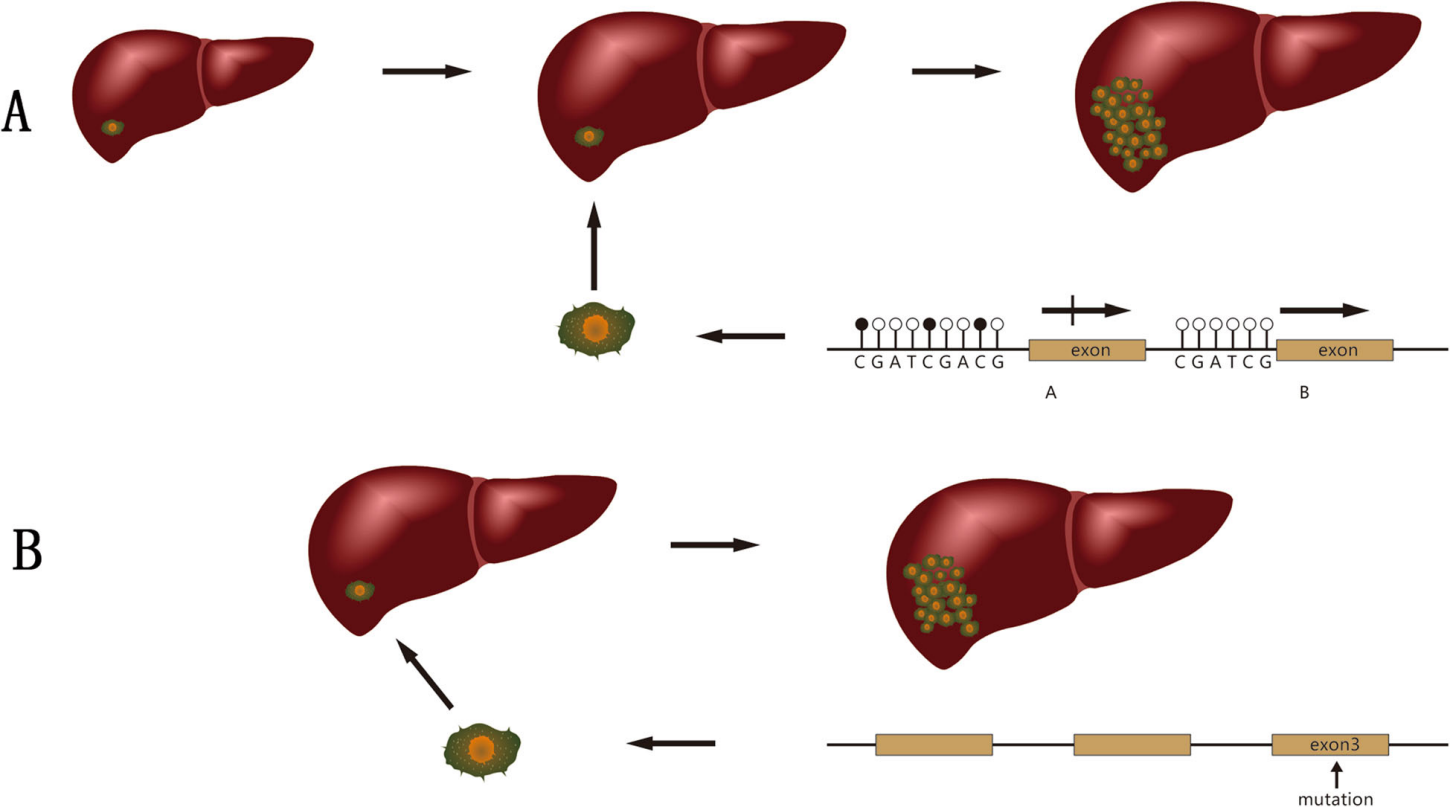
Two mechanisms of hepatoblastoma formation. **a**: Hepatic differentiation is blocked during the embryonic stage, and DNA methylation stays in the early stages of liver differentiation. Incompletely differentiated cells remain in the fetal liver after birth, and abnormal proliferation of the cells causes hepatoblastoma. **b**: The mutation of exon 3 of CTNNB1 gene in fetal liver cells causes β-Catenin accumulation, and abnormal cell proliferation leads to HB

In hematological tumors and most adult solid tumors, including liver cancer, TET gene mutations leading to loss of gene function and reduced 5hmC content are important causes of tumorigenesis [79–81] and indicate a poor prognosis [82]. TET acts as a TSG. However, in HB, both TET and 5hmC increase, which indicates that the tumorigenic mechanisms of TET and 5hmC in HB and adult tumors are different. Data indicate that during embryonic development, TET enzymes are highly expressed and inhibit differentiation [83, 84]. Therefore, we can speculate that the occurrence of HB is due to the obstruction of liver differentiation in the early stage of differentiation (Fig. 2). The origin of HB is the incompletely differentiated hepatocytes remaining in the liver of children. This is consistent with the conclusions obtained from a whole-genome methylation scan of HB [17], and it can also explain why HB includes many different histological types of cells.

## Diagnosis and treatment of DNA methylation aberrations

DNA methylation changes, as a general change in tumor cells, can cause tumor cells to show specific changes that make them different from ordinary cells. In the initial diagnosis of HB, imaging screening and biopsy-based pathological diagnoses are mainly used. These methods are more reliable and specific than other methods. DNA methylation can be used as an index for screening for tumor recurrence and metastasis during postoperative review and as an index for judging prognosis. According to the literature reports, the RASSF1A, UHRF1, MT1G, and NDRG2 promoter regions are hypermethylated. The degree of methylation is related to postoperative recurrence and distant metastasis and can be used as a marker for diagnosis and prognosis [31, 41, 54, 73, 85].

For the treatment of DNA methylation aberrations, because DNA methylation is achieved by the function of DNMTs, DNMTs are a therapeutic target for DNA demethylation and restoration of TSG functions. The most studied demethylating drugs at present are mainly DNMT inhibitors [86]. The mechanism of antitumor action of DNMT inhibitors is mainly to inhibit the activity of DNMTs so that the duplicated DNA is not methylated, and all alleles would be demethylated so that the expression of TSGs would be restored and they could play their role in inhibiting tumor growth, achieving antitumor therapy [87]. Currently, the most widely used DNMT inhibitors are decitabine (5-aza-2′-deoxycytidine) and azacytidine [88], but there is no clinical information on the treatment of HB with either drug.

## Conclusion and prospective directions

HB is a tumor that proliferates because hepatocyte differentiation is blocked in early development. Mutations of the CTNNB1 gene can be detected in most tumors, and DNA methylation plays an important role in the occurrence and development of tumors. Hypermethylation reduces the expression of specific TSGs, while global hypomethylation increases the expression of other genes, promoting tumor development through various pathways. The dysfunction of a variety of DNA methylation regulatory enzymes plays a key role in the hypermethylation of TSGs and the hypomethylation of oncogenes, especially the UHRF1 protein.

According to the review in this article, DNA methylation can affect the formation and metastasis of hepatoblastoma through different cell signaling pathways, and the metastasis of hepatoblastoma is an important factor affecting the prognosis. Therefore, further research on the mechanism of DNA methylation in hepatoblastoma formation and metastasis has important basic and clinical significance.

At present, various tumor cells show global hypomethylation and hypermethylation of specific TSGs. It is speculated that this wide range of methylation changes is accomplished by DNA methylation regulatory enzymes. Therefore, further research on the regulatory mechanism of DNA methylation regulatory enzymes and their role in tumor development should be a future direction. Second, the mechanism by which DNA methylation regulatory enzymes enhance the methylation of TSGs and reduce the methylation of other genes and whether other proteins play a role in this process needs to be further studied. Third, the main factors influencing the long-term prognosis of HB are vascular invasion and distant metastasis [89]. The methylation level of multiple TSGs is closely related to vascular invasion and distant metastasis, which can be studied with large sample sizes.

## Data Availability

Not applicable.

## Abbreviations

APC: APC regulator of WNT signaling pathway
CITED1: CBP/P-300 interacting transactivator 1
CK1: Casein kinase 1
CTNNB1: Catenin beta 1 gene
DMR: Differentially methylated region
DMS: Differentially methylated sites
DNMT: DNA methyltransferase
GPC3: Glypican 3
GPR180: G protein-coupled receptor 180
GSK3β: Glycogen synthase kinase 3 beta
HB: Hepatoblastoma
Hh: Hedgehog
HHIP: Hedgehog-interacting protein
IGF: Insulin-like growth factor
IGFBP3: Insulin-like growth factor-binding protein 3
JAB1: Jun activating binding protein 1
JAK/STAT: The Janus kinase-signal transducers and activators of transcription
LGR6: Leucine-rich repeat-containing G protein-coupled receptor 6
LOI: Loss of imprinting
Mst: Macrophage stimulating
MST1R: Macrophage stimulating 1 receptor
MT1G: Metallothionein-1G
NDRG2: N-Myc downstream regulated gene 2
NR5A2: Nuclear receptor subfamily 5 group A member 2
OCIAD2: OCIA domain containing 2
PARP6: Poly (ADP-ribose) polymerase family member 6
PLAG1: Pleomorphic adenoma gene 1
RASSF1A: Ras association domain family member 1A
SOCS1: Suppressor of cytokine signaling 1
SSI-1: STAT-induced STAT inhibitor-1
TET: Ten-eleven translocation
TGF-β2: Transforming growth factor-β2
TSG: Tumor suppressor gene
YAP/TAZ: Yes-associated protein/Tafazzin
